# The Effects of Graded Levels of Calorie Restriction: XX. Impact of Long-Term Graded Calorie Restriction on Survival and Body Mass Dynamics in Male C57BL/6J Mice

**DOI:** 10.1093/gerona/glad152

**Published:** 2023-06-24

**Authors:** Sharon E Mitchell, Jacques Togo, Cara L Green, Davina Derous, Catherine Hambly, John R Speakman

**Affiliations:** School of Biological Sciences, University of Aberdeen, Aberdeen, UK; School of Biological Sciences, University of Aberdeen, Aberdeen, UK; School of Biological Sciences, University of Aberdeen, Aberdeen, UK; School of Biological Sciences, University of Aberdeen, Aberdeen, UK; School of Biological Sciences, University of Aberdeen, Aberdeen, UK; School of Biological Sciences, University of Aberdeen, Aberdeen, UK; Shenzhen Key Laboratory of Metabolic Health, Center for Energy Metabolism and Reproduction, Shenzhen Institutes of Advanced Technology, Chinese Academy of Sciences, Shenzhen, P.R. China; State Key Laboratory of Molecular Developmental Biology, Institute of Genetics and Developmental Biology, Chinese Academy of Sciences, Beijing, P.R. China; China Medical University, Shenyang, Liaoning, P.R. China

**Keywords:** Autoregulation, Energy balance, Lifespan extension

## Abstract

Calorie restriction (CR) typically promotes a reduction in body mass, which correlates with increased lifespan. We evaluated the overall changes in survival, body mass dynamics, and body composition following long-term graded CR (580 days/19 months) in male C57BL/6J mice. Control mice (0% restriction) were fed ad libitum in the dark phase only (12-hour ad libitum [12AL]). CR groups were restricted by 10%–40% of their baseline food intake (10CR, 20CR, 30CR, and 40CR). Body mass was recorded daily, and body composition was measured at 8 time points. At 728 days/24 months, all surviving mice were culled. A gradation in survival rate over the CR groups was found. The pattern of body mass loss differed over the graded CR groups. Whereas the lower CR groups rapidly resumed an energy balance with no significant loss of fat or fat-free mass, changes in the 30 and 40CR groups were attributed to higher fat-free mass loss and protection of fat mass. Day-to-day changes in body mass were less variable under CR than for the 12AL group. There was no indication that body mass was influenced by external factors. Partial autocorrelation analysis examined the relationship between daily changes in body masses. A negative correlation between mass on Day 0 and Day +1 declined with age in the 12AL but not the CR groups. A reduction in the correlation with age suggested body mass homeostasis is a marker of aging that declines at the end of life and is protected by CR.

Recent World Health Organization statistics suggest the average life expectancy of the global population in 2019 was 73.3 years; however, a healthy life expectancy was nearly 10 years lower at 63.7 years (1). Consequently, with our increasing aging population comes the burden of age-related problems. Calorie restriction (CR) is the most reliable method of reducing the effect of age-related health issues and extending lifespan over a range of species, from yeast to mammals (2–4). As such, CR is a powerful tool for aging research with intensive investigations ongoing to understand the mechanisms behind CR-dependent extension of health and lifespan (5,6).

A short-term, moderate level of CR (~20%–40% restriction avoiding malnourishment) typically promotes many beneficial health effects such as reduced adiposity, improved lipid profiles, glycemic markers, and blood pressure as observed in rodents, nonhuman primates, and humans (4,7,8). In rodents, the increase in mean lifespan correlates positively with the level of CR. On average, a 40% CR resulted in a 30% increase in lifespan above that of ad-libitum controls (9). Whether CR is a potential intervention that can be utilized to prolong the life of humans remains debated (10,11).

Many factors affect the CR response, that is, age and/or body mass at the start of CR protocol and duration (12,13). Furthermore, the lifespan-lengthening response to CR is not universal across all mouse strains and in some cases, life shortening has been reported (14–16). Unraveling the mechanisms behind the anti-aging effects of CR is the first step to harnessing the power of CR to develop CR mimetics capable of counteracting the deleterious effects of aging in humans. Over a series of previous papers, we have extensively characterized the response to short-term (3 months; STCR), graded CR (from 10% to 40%) in male C57BL/6J mice from the whole-body phenotype (17), endocrine response (18), to behavior (19), multitissue transcriptomics (20), and metabolomics (21,22).

When animals are initially placed under restriction, they face an immediate shortfall between their energy demands and their energy intake. They respond to this imbalance by withdrawing stored energy from both fat reserves and lean tissues, increasing their digestive efficiency to extract more energy from the ingested food, linked to the expansion of some components of the alimentary tract (17), reducing their physical activity and energy expenditure (23) and lowering their body temperature (24). Reductions in lean tissue led to reductions in energy demands (25) and there may also be tissue-specific reductions in metabolic rate (26). We previously showed that it takes around 25–30 days for mice to resume energy balance—as defined by regained stability in their body mass and gross composition measured by dual-energy x-ray absorptiometry analysis (DXA), and this time was independent of the level of restriction (17). Thereafter, body mass and gross composition remain approximately stable until around 90 days of restriction (17). Whether there are further progressive changes as the duration of the restriction is extended beyond 90 days currently remains unknown.

In addition to the overall trends in body mass over time, there are also day-to-day fluctuations. These fluctuations result from the day-to-day imbalance between energy intake and expenditure and hence the need to store or withdraw energy—either as glycogen or fat. If an animal expends more than it consumes on any particular day, its weight will decline. The animal may compensate by then driving itself into a positive balance the next day to reverse the weight loss. Hence, it is anticipated that there will be a negative correlation between weight change on any particular day and the following day. These regulatory compensations may extend over multiple days depending on the magnitude of the changes on any particular day. In humans, the compensatory regulation seems to happen after 3–4 days rather than after 1–2 days (27,28). Few studies have addressed this pattern of autoregulation in energy balance and body weight over time, and none that we are aware of has addressed how such regulation changes with age or CR. In theory, animals fed ad libitum can respond to an energy imbalance on 1 day by changing both their food intake and energy expenditure the next day. For mice under CR where the ration of food is fixed, the available options to sustain stability are reduced to manipulating only expenditure. This may enforce in such restricted animals, a much greater stability, and this greater stability in day-to-day energy balance may be an instrumental factor related to their longevity. Yet how the regulatory feedback system in energy balance is affected by CR remains unknown.

Moreover, how the altered balance between fat and lean tissue links to survival and longevity is disputed. Although some have pinpointed the reduction in fat mass as a key factor to predict life extension under CR (29,30), others have conversely suggested that maintenance of fat mass is vital for survival (31). Fat-free mass also plays an active and passive role in the long-term regulation of body mass (32). With age comes a progressive loss of muscle mass, known as sarcopenia, with changes in muscle reported from 12 months in mice (33). Sarcopenia may be disadvantageous with respect to general health. This suggests composition changes may play important roles in the long-term consequences of CR treatment, yet our understanding of the progressive lifetime changes in composition is rudimentary.

This paper presents a comprehensive analysis of the survival and dynamic changes in body mass/composition in response to graded levels of long-term (19 months) CR (LTCR) with an analysis focusing on the temporal patterning and autocorrelation of body composition. These responses are in part compared to the changes observed in the same parameters after 3 months of restriction (STCR) which was started at the same age in the same sex and strain.

## Materials and Methods

### Animals, Ethics, and Diet

All procedures, carried out under Home Office License (PPL60/366), were reviewed and accepted by the University of Aberdeen Animal Welfare and Ethical Review Body. Studies were compliant with the Animals (Scientific Procedures) Act 1986 and in accordance with the ARRIVE Guidelines (34). The rationale for the work followed that of our STCR study (84 days/3 months) described in detail elsewhere (17). The current study was designed to characterize the effects of long-term graded CR (LTCR; 588 days/19 months). CR was initiated at 140 days/5 months old and mice were sacrificed at 24 months of age, which is considered to be equivalent to ~20 and ~70 years old in human terms, respectively (35).

Sixty-four male C57BL/6J mice (Charles River, Ormiston, UK) were acclimated to single housing for 2 weeks with free access to food and water. Single housing was used because the implanted telemetry devices cannot function with more than 1 mouse per cage. This procedure also allowed the accurate provision of the CR dosage at the individual level and avoided any within cage aggression. During baseline, a 12-hour ad-libitum (12AL) feeding regime was introduced where food was provided only in the hours of darkness. We previously characterized the effects of STCR using both 12AL and 24-hour ad-libitum (24AL) fed mice. We found in general no differences across a whole range of metrics between 12AL and 24AL mice including energy intake and body composition (17). 12AL feeding prevented daytime eating in controls (36,37) and hence avoided any consequential effects of the 24AL mice eating pre-cull. For LTCR study, we chose 12AL as the controls rather than 24AL so that all mice were in a similar unfed state for hormonal and metabolic comparisons. In addition, CR animals are known to gorge upon provision of their daily food ration, resulting in ~20+ hours of daily fasting. This fasting may contribute to the observed lifespan impact of CR protocols (38,39). The 24AL access to food in controls may confound comparisons with the CR groups due to the multitude of hormonal, metabolic effects of eating; therefore, the 12AL group had zero restriction but extended fasting similar to the other CR groups. Prior to starting, CR mice underwent a 2-week baseline period during which several measurements were taken including DXA.

Mice were allocated to 5 groups initially matched for body mass: 12AL (*n* = 14), 10CR (*n* = 14), 20CR (*n* = 12), 30CR (*n* = 12), and 40CR (*n* = 12), which refers to the percentage level of restriction, that is, 30CR mice were fed 30% less their individual total daily calories measured over the 14-day baseline. Given the level of CR has a positive linear relationship to the magnitude of the lifespan extension, numbers were increased in the 12AL and 10CR groups in anticipation of higher mortality in these groups. CR was not implemented in a stepwise reduction manner. The CR mice were provided food rations on the floor of the cage to avoid difficulties accessing single pellets from the hopper.

Given the long-term nature of the study, diet formulations were modified slightly from that of the original study. Although the macronutrient composition remained the same (70% carbohydrate, 20% protein and 10% fat (kcal%), the diet we used D12450H had lower sucrose (17% vs 35%) than D12450B used in the STCR study; Research Diets, New Brunswick, NJ, USA). All mice were fed D12450H over baseline and the 12AL, 10CR and 20CR groups over the CR phase. Essential to the beneficial effects of CR is the avoidance of malnutrition. Long-term changes in the 12AL food intake and, therefore, the exact balance of vitamin mix to exactly equate intakes of vitamins and minerals across the groups could not be anticipated. Thus, mice in the 30CR and 40CR groups were fed a diet matched to D12450H containing a 40% increase in the vitamin mix to prevent any nutrient deficiency (D13020504; Research Diets, NJ, USA). Individual CR food allocations were calculated from the average daily food intake over a 2-week baseline period where all mice were fed the same 12AL regime. Individual food pellets were cut to size and CR mice were fed daily, directly before the start of the dark phase. 12AL mice were provided with food in the hoppers at 1830 hours and any remaining food was removed at 0630 hours. Body mass was measured daily immediately prior to feeding, from ~1700 hours.

### Body Composition

Body composition was determined using DXA, GE PIXImus2 Series Densitometer (GE Medical Systems Limited, Chalfont St. Giles). Fat mass and fat-free mass were measured at 8 time points; 0, 28, 56, 84, 168, 252, 365, and 580 days (0, 1, 2, 3, 6, 9, 12, and 19 months) of CR. Mice were anesthetized with isoflurane for the duration of the scan. Data were corrected using an equation specific to the DXA machine (40).

### Euthanasia/Pathology

Although changes in body mass give an indication of the animal’s health status, conditions such as tumors or ascites may falsely increase body mass; therefore, body condition scores and humane endpoints for aging animals were agreed with University Veterinarians at the onset of the study (41,42). Body condition was scored between 1 and 5 with 3 being the optimal condition, 1 emaciated, and 5 obese (42). Mice were checked twice daily, at lights on and off, and any signs of pain or stress, or detraction from normal behavior noted. In general, a humane endpoint was determined if the body condition score was <2; an unexpected weight loss was measured over 3 consecutive days; weight loss was >10% in less than 24 hours; or total weight loss exceeded 30%. Excessive weight gain/swollen abdomen in comparison to mice of the same group may indicate tumor. A necropsy examination was carried out for all mice euthanized during the study. Disease states were identified and categorized as neoplasia, age related (as age increases the risk also increases), or nonage related (risk does not increase with age) (41). Neoplasms were no further determined as malignant or benign.

### Statistical Analysis

All statistical tests were conducted in RStudio 2022.02.2 + 485 using R Statistical Software (v4.1.1 (43)). The ggplot2 R package was used for data analysis and visualization (44). This study was designed to investigate the response to graded CR and to elucidate the mechanisms involved in improving health span and increasing lifespan. Over the course of the study, 31 of the starting 64 animals were euthanized prior to the scheduled endpoint of 24 months. To include all data for all mice introduces a bias to the data set with changes in the selection of mice. Given the research question was to elucidate how CR promotes longevity, 1 approach was to include the 33 survivors only. However, as this potentially also introduces a bias, if euthanized mice were already different at the start, where appropriate the starting 64 mice were analyzed as well. This will be clearly stated in text what analysis pertains to which group. Survival was analyzed using the Kaplan–Meier method and Cox proportional hazards regression model. The point to inflection, when body mass stabilized, was calculated from fitted second-order polynomials (ie, stabilization point equals coefficient of *x* divided by twice the coefficient of *x*^2^: See (17) for derivation). The level of CR was treated as a continuous variable (0–40, with the 12AL controls represented by 0). The body composition responses to graded LTCR were analyzed using least-square linear regressions (α = 0.05). Body composition parameters were correlated with lifespan (Pearson’s correlation). Fat mass and fat-free mass values were taken from the most recent DXA scan. For the longitudinal DXA measures, a linear mixed model (estimated using REML and nloptwrap optimizer) was fitted to predict body mass, fat mass, or fat-free mass. Day and level of CR were both treated as continuous variables and included as covariates with mouse ID as a random effect. Standardized parameters were obtained by fitting the model on a standardized version of the data set. Ninety-five percent confidence intervals (CIs) and *p* values were computed using a Wald *t*-distribution approximation.

A *p* value of <.05 was considered statistically significant and adjusted using the Bonferroni method. Paired *t* tests were used to compare individual body composition parameters between baseline and the end of study.

### Body Mass Dynamics

Mouse body masses were measured every day throughout the study. Using the autocorrelation function, patterns in body mass change were identified, that is, how does the present body mass influence future body mass. The change in body mass was calculated between Day 0 and Day +1 for all day-to-day pairs of body mass. The level of variation in the day-to-day changes was explored between the different groups by making all the changes positive and then taking the average change in body mass for each individual and comparing across groups using ANOVA (followed by post hoc Tukey comparisons). We explored whether the day-to-day changes were correlated across cages by computing the correlation coefficient for each pair of cages (*n* = 1 953 correlations) over a period spanning 80 days when *n* = 63 mice were alive. The 80-day period was free of other procedures potentially affecting body mass such as glucose tolerance test (GTT) during when mice are fasted overnight. The distribution of these correlations reflects whether change in body mass over given days was driven by events external to the cages (eg, disturbance or room temperature fluctuations). If so, the distribution would be positively skewed, as all mice would be affected. However, if each animal varied its body mass independent of the others with no external cues, the distribution would be expected to be symmetrical around zero.

To explore the correlation of the day-to-day changes in body mass over time, we performed an autocorrelation analysis. This analysis effectively asks if there is any relationship between the weight change on a given day (d0 to d1) and subsequent days d1 to d2, d2 to d3, etc. If weight fluctuates at random, we would expect no correlation across days, but if weight is regulated in some way, then a correlation across days would be anticipated. We performed the analysis with lags of up to 10 days. This analysis showed that there was only a significant autocorrelation with a lag of 1 day. In other words, the change in mass on a given day was related to the change in mass the next day but was unrelated to mass changes after that. To explore how that autocorrelation changed with age in the different groups, we split the day-to-day mass change data into 50-day bins, that is, Days 0–50, 51–100, etc., and then for each individual looked at the correlation with a lag of 1 day as a function of age and also the level of restriction.

## Results

### Survival

**Figure 1.**
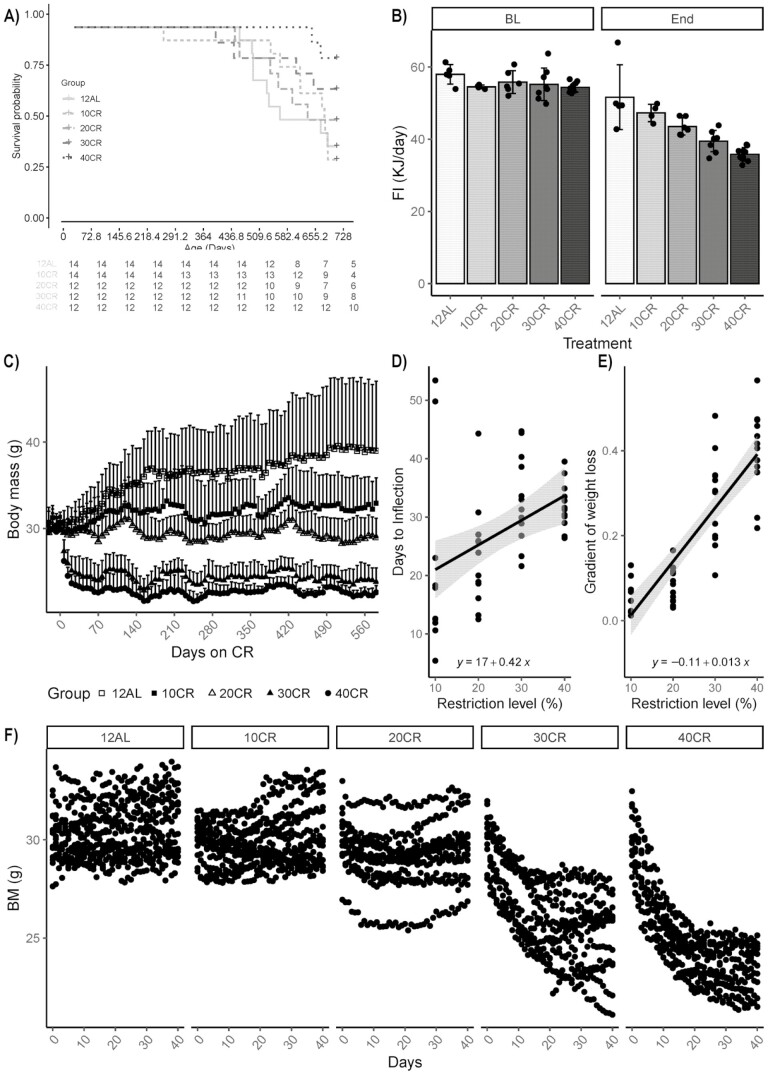
Effects of graded calorie restriction (CR) on lifespan, food intake (FI), and body mass (BM) of male C57BL/6J mice. (A) Kaplan–Meier survival curves for male mice fed 12-hour ad libitum (12AL) or 10%, 20%, 30%, or 40% reduction from baseline food intake (10CR, 20CR, 30CR, and 40CR, respectively). Age in days is shown along the *x-*axis. CR began at 140 days of age (5 months). *n* survivors for each group are denoted over the course of the study. (B) Daily FI measured over baseline (BL) and final week of study (End). (C) Growth curve of BM plotted against time on CR in days. (D) Time taken (days) for weight loss to stabilize. Inflection points were calculated from fitted polynomial curves to the weight change from Days 1 to 40 inclusive. (E) The rates of weight loss of mice on CR, calculated from the sum of the coefficients in *x* and *x*^2^ of the second-order fitted polynomial to the BM over the first 40 days of CR. (F) BM changes over the first 40 days of graded CR. Data for B and C include 33 survivors only (*n* = 5 for 12AL, *n* = 4 for 10CR, *n* = 6 for 20CR, *n* = 8 for 30CR, and *n* = 10 for 40CR). Data for D, E, and F include all 64 mice, *n* = 14, for 12AL and10CR, *n* = 12 for 20CR, 30CR, and 40CR.

A total of 33 out of the 64 animals remained alive at the 24-month-old endpoint (728 days of age/588 days of CR). As anticipated increasing CR had a favorable effect on survival (likelihood ratio test = 9.89, df = 4, *p* = .042), significant only in the 40CR mice (*p* = .02, Cox proportional hazards regression model; Figure 1A). Median survival was 646 days in the 12AL controls, with a survival rate of 35.7% at 24 months. Although the survival rate was lower in the 10CR, 28.6%, a graded improvement in survival in relation to 12AL was observed with the 20, 30, and 40CR mice (14.3%, 31%, and 47.6%, respectively; Figure 1A).

### Disease States

An “assumed cause” of death was ascertained from a necropsy examination of the tissues of all mice (the 31 euthanized and the 33 that reached 24 months). Disease incidence was categorized as neoplasia/cancer, age related, and non-age related. Examinations revealed neoplastic disorders were the primary cause of death, found in 19 of the 31 mice euthanized (Supplementary Table 1). Of the 6 types of cancer/neoplasia identified, the liver was the most common, 53% of all cancers. Individual findings from the necropsies are reported in Supplementary Table 2. Although cancer incidence was the lowest in the 40CR mice pre-24 months scheduled cull (1 from 12, Supplementary Table 1), at 24-month cancer/neoplasia was the highest (4 of the remaining 10). This indicates CR does not prevent the initiation of cancer but the increase in probability of survival in the 40CR was due to a slower progression of cancer. Nonage-related assumed causes of death were attributed to not eating with weight loss and no obvious disease state (2 cases), 1 case of distended intestines and hardened epididymal fat pad, 1 of hernia, and 1 of enlarged lungs.

Body mass changes are often regarded as an indicator of health in mice. Taking the body mass change over the 7 days prior to early euthanasia, the body mass of 8 mice was within ±0.5 g, a loss >0.5 g was recorded in 16, and a gain >0.5 g in 7. Body mass changes prior to death ranged from a 3.04 g gain to a 5.82 g loss. These particular mice were found to have a liver tumor (3.04 g gain) and became chronically hyperglycemic post-experimental GTT and stopped eating (5.82 g).

### Food Intake

The baseline food intake averaged over the 33 surviving animals was 55.4 ± 3.0 kJ/d (1-way ANOVA *F*_(4, 28)_ =1.43, *p* = .25; Figure 1B). The graded restrictions were calculated based on an individual’s intake during baseline. However, at the end of study, the level of restriction in the surviving animals was lower at 8%, 16%, 24%, and 31% for the nominal CR groups 10–40CR, respectively, when calculated in comparison to the average intake of the 5 remaining 12AL fed mice (52.2 ± 10.2 kJ/d; least-square regression *R*^2^ = 0.69, *p* < .0001; Figure 1B). Although energy intake in the 12AL group decreased over the period of study, this was not significant (paired *t* test; baseline vs final food intake; *t* = 2.84, *p* = .28).

### Body Mass

Body mass was weighed daily throughout the study generating a unique data set with a possible 37 440 mass points (64 mice, 585 days CR) if they had all survived the complete duration. In reality, we collected 33 289 data points. A dynamic phase of body mass change was observed after the introduction to CR as mice strive to reach a new energy balance (Figure 1C). This dynamic stage was analyzed in all 64 starting mice. Average body mass across mice at the start of CR (Day 0, age 140 days) was 30.2 ± 1.1 g (1-way ANOVA *F*_(4,59)_ = 0.59, *p* = .67; Figure 1C and F). Body mass loss was rapid in the 30CR and 40CR groups and body mass significantly lower relative to the 12AL controls by Days 3 (40CR, 2.04 ± 1.5 g loss; *t* = 3.33, *p* = .009) and 4 (30 CR, 1.97 ± 1.4 g loss; *t* = 3.53, *p* = .004; Figure 1F). Although an initial loss of body mass was observed in some mice of the 10CR and 20CR groups, comparisons between mice in 12AL found no significant difference over the 40 days (*t* = 0.83, *p* = 1.0; and *t* = 2.29, *p* = .22, respectively).

The point of inflection, when body mass stabilized, was calculated from fitted second-order polynomials (*n* = 47, 3 mice in the 10CR group were omitted as gained weight or did not stabilize over the 40 days; Figure 1D and F). The time taken to stabilize body mass took longer with increasing CR level (least-square regression *R*^2^ = 0.19, *p* = 0.002; Figure 1D). The time at which body mass stabilized was 21 ± 16 days for the 10CR, 23 ± 9 days for 20CR, 33 ± 8 days for 30CR, and 32 ± 4.0 days for the 40CR. Averaged across all CR groups, this was 27.5 ± 10.8 days (Figure 1D). Individual variation in time to reach a stabilized body mass was high in the 10CR (ranging from 5 to 53 days). With increasing CR level, the response was more similar among individual mice and the within-group variation was reduced (40CR ranged from 26 to 39 days; Figure 1D). The gradient of weight loss on Day 1 was linearly related to the level of restriction *y* = 0.013*x* – 0.11, *R*^2^ = 0.73 (Figure 1E).

### Body Composition

**Figure 2.**
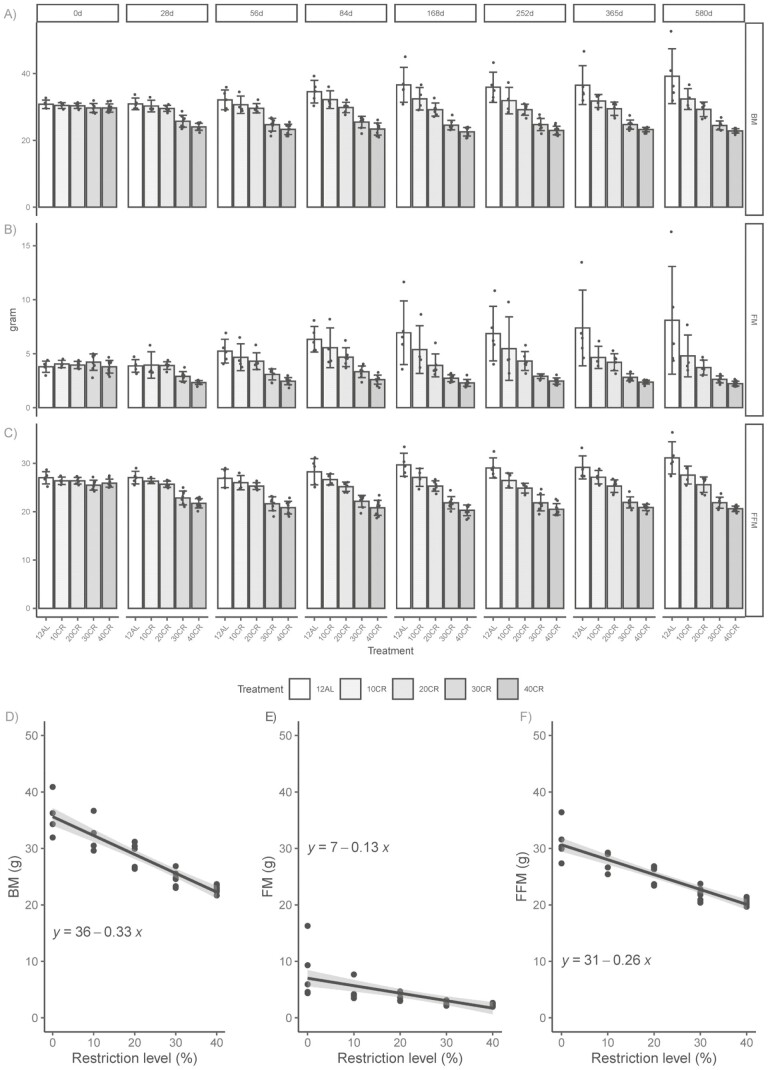
Body composition of male C57BL/6J mice measured at 8 time points points over 580 days/19 months of graded calorie restriction (CR). (A) Body mass (BM), (B) fat mass (FM), and (C) fat-free mass (FFM) measured in male C57BL/6J mice fed 12AL (12-hour ad libitum), or restricted by 10%–40% of their baseline food intake (10CR, 20CR, 30CR, and 40CR) from the age of 140 days/5 months. Body composition changes over the 580 days of CR with linear regressions equations shown for (D) BM, (E) FM, and (F) FFM. Data shown as mean ± *SD* for the 33 surviving mice. *n* = 5 for 12AL, *n* = 4 for 10CR, *n* = 6 for 20CR, *n* = 8 for 30CR, and *n* = 10 for 40CR. Data shown as mean ± *SD*, * indicates significant difference from 12AL, *p* < .05. *SD* = standard deviation.

Body composition (body mass, fat mass, and fat-free mass) was measured using DXA. Average body mass at baseline DXA was 30.07 ± 1.2 g, fat mass 3.96 ± 0.6 g, and fat-free mass 26.12 ± 1.0 g (Figure 2A–C). Changes in body mass, fat mass, and fat-free mass over 8 time points in the 33 surviving mice were analyzed using a linear mixed model. In all cases, the level of CR was highly significant and interacted with time. Body mass: Day (*t*_258_ = 6.14), CR level (*t*_258_ = −8.62), and an interaction between the 2 (*t*_258_ = −8.31) were statistically significant *p* < .001. Fat mass: Day (*t*_258_ = 5.38), CR level (*t*_258_ = −7.05) and an interaction between the 2 (*t*_258_= −7.23) were statistically significant *p* < .001. Fat-free mass: Day (*t*_258_ = 5.13), CR level (*t*_258_ = −9.02) and an interaction between the 2 (*t*_258_ = −7.42) were statistically significant *p* < .001.

A gain in body mass was recorded in the 12AL group over the study, 8.41 ± 8.1 g, *n* = 5 (Figure 2A and Supplementary Table 3). C57BL/6J mice are known to show a high level of phenotypic variability and the coefficient of variance (CV%) was high, 20.95%, with a body mass range from 31.94 to 52.69 g. This significant gain in body mass was reflected by similar increases in fat mass (4.3 ± 5.2 g) and fat-free mass (4.11 ± 2.9 g; Figure 2B and C and Supplementary Table 3). At the end of the study, a significant linear decrease in body mass with increasing level of CR was observed (least-square regression *R*^2^ = 0.72, *p* < .0001; Figure 2D). The loss in body mass relative to the 12AL was −17%, −25%, −38%, and −42% in the 10, 20, 30, and 40CR groups, respectively (Figure 2A).

Linear reductions in fat and fat-free mass with increasing level of CR were also observed (least-square regression *R*^2^ = 0.45, *p* < .0001, *R*^2^ = 0.83, *p* < .0001; Figures 2E and F, respectively). Although both the loss of fat and fat-free mass constitute the overall body mass change, the slope from fat-free mass followed closer to that of body mass. Fat mass in the 12AL mice was 8.09 ± 4.99 g (range 4.36–16.28 g) at the end of the study. Relative to the 12AL controls, a graded loss in fat mass was measured in the 10–40CR (4.8 ± 1.94, 3.72 ± 0.7, 2.63 ± 0.32, and 2.23 ± 0.22 g equivalent to −41%, −54%, −68%, and −72%, respectively; Supplementary Figure 1A and C and Supplementary Table 3).

Similar to the 12AL controls, individual variation was also high in the 10CR mice. In fact, 3 of the 4 surviving mice in the 10CR group compensated for the reduction in calories with gains in body mass, fat mass, and fat-free mass compared to their baseline mass (Supplementary Figure 1). The pattern of body composition changes in mice at the higher CR levels, 30CR and 40CR, was distinctly more dramatic than the 10 and 20CR (Figure 2). Following 580 days of CR, body mass loss was −18% in the 30CR group and −23% in the 40CR groups compared to baseline body mass (Supplementary Table 3). As a repercussion of the fat mass increases in the 12AL, changes in fat mass compared to controls were −67% and −72% in the 30CR and 40CR, respectively, after 580 days of CR (and Supplementary Figure 1 and Supplementary Table 3). The amount of fat lost was strongly related to fat mass at the start of the study in the 30 and 40CR groups (Supplementary Figure 2A). Fat-free mass loss was −30% and −34% relative to 12AL at the end of the study.

To understand the relationship between the changes in body composition, correlations were plotted separately for the 22 CR mice euthanized and the 28 survivors. A significant relationship was found between fat mass and fat-free mass, for both groups (*R* = 0.71, *p* = .0002 and *R* = 0.77, *p* = .0001; Supplementary Figure 2B). However, a higher number of survivors (SV) were found in the bottom left quadrant with the highest loss of fat mass and fat-free mass.

### Body Mass Dynamics in Time

**Figure 3.**
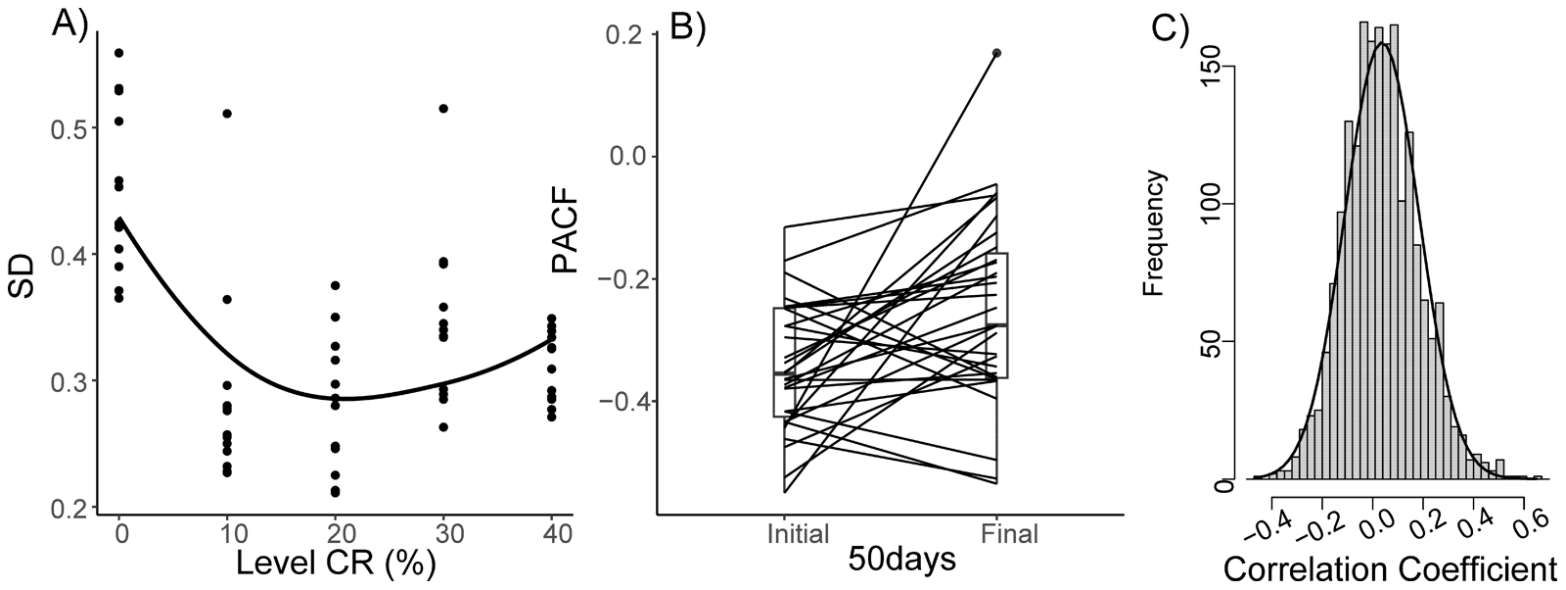
(A) Individual day-to-day differences in body mass of mice under graded calorie restriction (CR). Daily differences were plotted over Days 50–250 and standard deviation (*SD*) plotted. Restriction level refers to reduction in baseline ad-libitum food intake 10%, 20%, 30%, and 40%. 0 refers to the control mice fed 12-hour ad libitum. *n* = 63. (B) Paired data plots of the partial autocorrelation (PACF) with a lag of 1 day in the first 50 days (Initial) compared to the final 50 days (Final) in the 31 mice euthanized over the study (*n* = 9, 8, 6, 4, and 2 for the 12AL, 10–40CR, respectively). (C) Frequency distributions of correlation coefficients for individual daily changes in body mass. Changes were calculated from Days 180 to 260 of CR.

Body mass in all groups stabilized once energy balance was reached (27.5 ± 10.8 days averaged over all groups; Figure 1C and D). Exploring the dynamics in daily mass over Days 50–250, when all bar 1 mouse was alive, revealed the variance in daily body mass was different across the restriction levels (*R*^2^ = 0.14, *F*_(1,61)_ = 9.69, *p* = .003; Figure 3A) with higher variation at the lowest levels of restriction, and particularly so among 12AL fed mice (Supplementary Figure 3).

**Figure 4.**
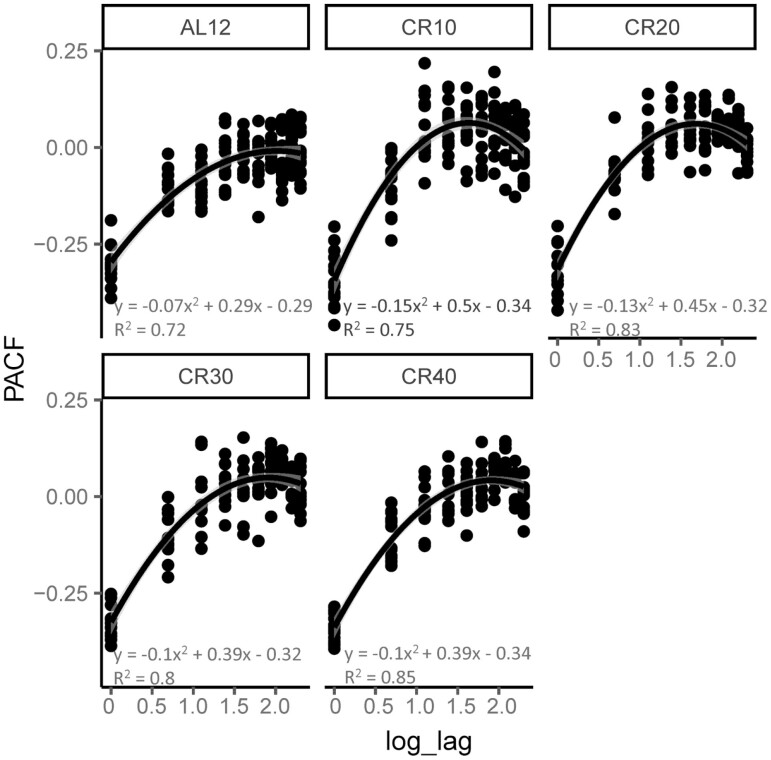
Partial autocorrelation patterning over lags of 1–10 days in mice on graded calorie restriction (CR). CR10, CR20, CR30, and CR40 refer to mice restricted by appropriate % of their own baseline food intake. Control AL12 mice were given ad libitum access to food for 12 h/d.

We explored the autocorrelation in the patterns of day-to-day change in body mass using a partial autocorrelation function analysis. The autocorrelation patterns were then pooled across the individuals in each level of restriction (Figure 4). The observed patterns were very similar across the different groups (Figure 4). In all cases, there was a strong and significant autocorrelation with a lag of 1 day. Thereafter, at all higher lags, the relationships were not significant. Examples of the day-to-day correlations with lags of 1, 5, and 10 days for individual mice are shown in Supplementary Figure 4.

### Body Mass Dynamics With Age

**Figure 5.**
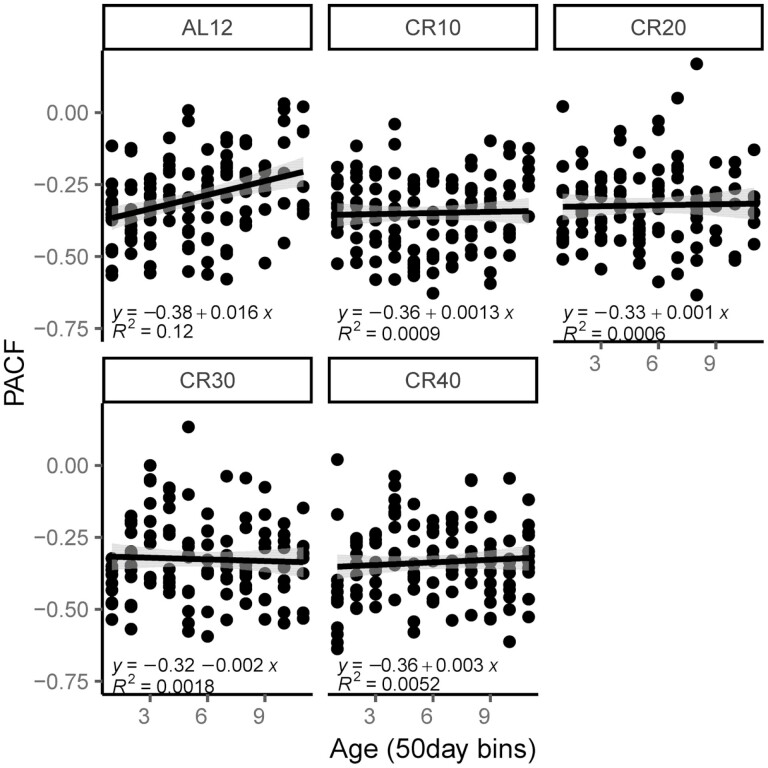
Body mass dynamics with age. Age on the *x*-axis represents 50-day blocks over the restriction period was plotted against partial autocorrelation (PACF). Linear relationships are shown. Calorie-restricted mice (CR) were fed 10%, 20%, 30%, and 40% less food than baseline intake (CR10, CR20, CR30, and CR40). Control mice were fed 12 hour ad libitum (12AL). Only the pattern at 12AL was significant.

To explore how the regulation of body mass changed with age, we divided the daily body mass data into 50-day blocks and then plotted the correlation between weight change on Day (*n*) with that on Day (*n* + 1) as a function of age (Figure 5). For the mice in the 12AL group, there was a linear decrease in the strength of the correlation with age (*R*^2^ = 0.12, *p* < .05). In contrast for all the mice under restriction, there was no trend in the correlation with age (*R*^2^ = 0.01, 0.01, 0.02, and 0.05 for 10CR,10CR, 30CR, and 40CR, respectively, all *p* > .05). Comparing the correlation with a lag of 1 day between the first 50 days and the 50 days immediately prior to death showed that the correlation with a lag of 1 day declined significantly as the mice approached death (*t*_30_ = −2.79, *p* = .009; Figure 3B).

To explore if body mass changes reflected some external factor, the changes in body mass from days 180 to 260 were correlated across all mice (*n* = 63 mice, 1 953 correlations). The frequency distribution was bell shaped and centered on zero indicating individuals were basically changing at random with respect to other individuals caged in the same room (Figure 3C).

## Discussion

Aging is inevitable but remains poorly understood (45). With human lifespan increasing disproportionately to health span, there is a necessity to better understand the aging process and thereby prolong our health span (46). CR is well documented to delay the aging process, and reduce the risk of age-related disease, such as cancer and type II diabetes, thus extending health and lifespan in many species (2,3,47). Although the potential to prolong healthy living and prevent age-related disease by simply eating less food is attractive, if this is to be achieved in humans the response to CR needs to be fully characterized and the triggers behind its beneficial effects elucidated.

Over a series of previous papers, we have characterized the responses of male C57BL/6J mice to STCR (3-month long) and found a number of phenotypic responses which may affect lifespan. A reduction in fat mass and circulating adipokines, improved insulin/insulin-like growth factor signaling (IIS), with a reduction in major urinary proteins and size of accessory organs indicating reduced reproductive investment, were found to be important factors in the STCR response (17,18,48). In addition, a positive effect on autophagy and xenobiotic metabolism, with increased removal of senescent cells and protective role against cancer, was found (48,49). Little support was found for a role of sirtuins (22).

However, the mice from the STCR were still relatively young, ~240 days/8 months old, when these measurements were made. Many studies have also shown the health benefits of STCR (reviewed (50)) but with aging comes many alterations, be it metabolic, molecular, or biochemical in multiple systems (45). Hence, whether there are additional more progressive changes over time remains uncertain. Here we investigated longer-term graded CR (728 days/19 months: LTCR) with mice 24 months old at the end of the study. Although maximum survival is the ultimate marker for lifespan, response data from mice in the latter portion of the survival curve will reflect influences of senescence and reflect the age-dependent risk on survival (51).

Thirty-six percent of the 12AL controls were alive at the end of the study (728 days/24 months). This was slightly lower than the projected 50% survival rate of 720 days given by the supplier, Charles River, for male C57BL/6J. A graded response in survival in relation to higher levels of CR was recorded with 28%, 50%, 67%, and 83% of mice in 10CR, 20CR, 30CR, and 40CR alive at 24 months old. This pattern of greater survival at higher levels of restriction matches the positive linear response in lifespan in relation to higher levels of CR that has previously been reported (9). This contrasts previous work from the National Institute on Aging (NIA) in the same strain and sex, which suggested that lifespan was extended with 20CR but did not follow a graded increase with 40CR (52). In female C57BL/6J, 20CR also increased lifespan with no response found with 40CR. However, DBA/2J of both sexes responded in the anticipated graded manner 40CR (52). Again, this was unexpected given earlier reports that DBA mice were unresponsive to CR (53) or only elicited a mild response in comparison to C57BL/6J (54). The reasons for the laboratory-to-laboratory differences in response remain uncertain. One notable difference between the NIA study and our graded CR studies was housing conditions. While the NIA mice were grouped (3–4 per cage), our mice were single housed. Some have suggested that group housing in CR studies may evoke dominance and aggression leading to unequal food allocation and resulting, in some cases, in lifespan shortening (55). This idea was rejected based on lack of evidence of dominance (56). Using body mass as a dominance indicator, heavier, that is, dominant mice, would live longest whereas those obtaining less food, that is, the subordinates, would have a shortened lifespan; this was not the case (56). In addition, food competition would provoke variance in body mass between grouped mice, but the coefficient of variation was not increased compared to AL (56). In the end, however, the data from NIA fall within the cloud of data from studies across all rodents linking the level of restriction to lifespan extension (9) suggesting these observations are not incompatible with the overall trend that more restriction generally leads to greater life extension at least up to 65% restriction.

Laboratory-to-laboratory differences in the response to restriction have been noted previously (discussed (2)). These differences may reflect a myriad of factors including sex (52), the dietary composition (57), the exact feeding protocol (39), and the age at the start of restriction (12). Notable were the conflicting results of 2 independent CR studies on rhesus monkeys. Although the Wisconsin National Primate Research Center reported beneficial effects of CR on health span and an indication that lifespan was increased (58), the results were opposed by the U.S. NIA (59). Differences in diet composition and feeding regimes were cited as possible reasons behind the discrepancies (7). These incongruent responses highlight the complexity we will ultimately face in individualizing dietary restriction programs for humans (60).

Necropsy results here found neoplasm to be the primary killer of C57BL/6J male mice which agrees with previous research (41,61). The incidence of cancer in mice euthanized prior to 24 months old declined as CR level increased. However, the final dissection of mice alive at 24 months revealed neoplasia in a high percentage of the survivors; 40% of the 40CR group (4 of the 10), 38% (3 of 8) in the 30CR, and 17% (1 of 6) in the 20CR. From these results, we can conclude that as previously reported, CR delayed the progression of these tumors (62). In humans, cancer accounted for close to 10 million deaths worldwide in 2020 (63). The beneficial effects of CR, or alternatives to reduced calorie intake such as periodic fasting, are increasingly recognized as reducing morbidity, preventing malignancies, and improving mortality (64,65). Results from our STCR study found an altered hepatic gene expression indicating the protective effect of CR on tumor growth (48).

A number of physiological changes apparent in the LTCR study here were remarkably similar to that found in our previous STCR (17). The time at which weight loss stabilized was 29.3 days in the STCR study and 27.5 days in the LTCR. However, there was not such a clear gradation in body composition loss over the levels of CR as noted in the STCR. Here some of the mice in the 10CR group quickly recovered body mass and, in some cases, gained over the 19 months. Although the exact protocol of the STCR was followed with regard to feeding times and procedures, given the longer duration of this study, there was a small alteration to the diet. D12450B, used in the STCR study, contained 35% sucrose which, according to the manufacturers, may induce hypertriglyceridemia, insulin resistance, and fatty liver (Research Diets). To avoid this, we used D12450H containing 17% sucrose, with the carbohydrate substituted with an additional 13.5% corn starch and 3.4% maltodextrin. These minimal micronutrient changes are unlikely to have limited the weight loss in the 10CR mice. Recent research indicated varying dietary sucrose levels from 5% and 30% did not affect weight gain (66).

More likely to explain the differences from the STCR study was the high variation in body composition within the 12AL control and the 10CR diet groups. Despite being inbred and considered to have genetic uniformity, C57BL/6J mice are well known to display large variations in body mass, specifically fat mass, when fed a high-fat diet (67,68). Although this effect has been profusely examined with regards to diet-induced obesity, it appears that this variation is not diet specific and the characteristics of high or low weight gainers are evident after 6 weeks of low-fat diet (69). The significant increase in body mass in the control 12AL was mainly reflected by high variation in fat mass; a CV of 62.5%. In response to CR, variability in body mass was lower with increasing CR level, ie, in mice fed 40CR the CV was only 9.9%.

The changes in body composition were mainly reflected by changes in fat-free mass. Calculated from DXA, fat-free mass includes everything bar fat tissue, that is, organs, muscle, and bone mass. With age, one of the most prominent body composition changes is sarcopenia, a loss in skeletal muscle mass, decreased strength, and aerobic capacity (70). Sarcopenia is also closely linked to age-related losses in bone mineral density, basal metabolic rate, and an increase in body fat mass. A number of age-related issues, including type II diabetes, hypertension, and osteoporosis, are associated with sarcopenia, in which CR has been shown to attenuate (71). After 28 days of 40CR and 56 days of 30CR, there was no further loss of fat-free mass over the remaining time of the study suggesting a protective effect of CR. This is not to say that the body is not undergoing further remodeling between the tissues.

Fat tissue is more energy dense than lean (39.5 vs 17 kJ/g) (17,72). A higher loss of fat mass would ultimately release more energy to alleviate the energy deficit provoked by CR. Given the mice stabilized body composition changes from 84 days, the loss is not age related to the survivors. Liao and colleagues measured body composition by echo magnetic resonance imaging in over 300 mice and reported the maintenance of fat mass during CR projected a longer survival in 38 male and 33 female strains of recombinant inbred ILSXISS mice (15,31). None of the ILSXISS strains that showed an extension in lifespan reduced their fat mass. In contrast, the fat mass of the surviving 30CR and 40CR mice here was significantly lower compared to the 12AL. Taken together, our results indicate a relationship between fat mass loss and longevity.

The response of the different fat depots to CR may be an important factor in the variable longevity under 40% CR in 3 of the ILSXISS mouse strains (73). Fat mass can be separated into distinct depots, visceral or subcutaneous, also regarded as “bad” and “good” fat. Visceral fat is associated with several metabolic diseases, and the surgical removal in rats increased longevity (30). Although our results agree with fat mass reduction as a key mechanism of CR anti-aging effect, earlier studies argue against this (74,75). As an endocrine organ, fat tissue plays an important role as a messenger in the control of energy balance. Future papers will look more at the influence of specific fat depots and circulating adipokines.

### Body Mass Dynamics

Irrespective of the level of restriction, we found that mice only regulated their body mass over periods of about 24–48 hours. This was shown because correlations between mass change on a given day and their mass changes over subsequent days were not significant after more than 1 day had elapsed. This contrasts with the responses in food intake and physical activity in humans, which show corrective responses with a lag of 3–4 days but not 1–2 days (27,28). The reasons for the difference remain unclear, but an obvious possibility is the large difference in body size, meaning day-to-day fluctuations in mice are greater relative to the total budget and thereby need to be compensated more rapidly. Alternatively, a time constant of 3–4 days is suspiciously close to half of the weekly cycle of weekdays and weekends, and the correlations over this period may therefore just be an artifact of human working patterns.

Mice under higher levels of restriction showed less day-to-day variation in their body mass changes. This variance was particularly elevated in the 12AL mice compared to those under restriction. We anticipated this might be the case because such mice have 2 degrees of freedom to affect their body mass because their intake and expenditure are both variable, whereas mice under restriction can only influence their day-to-day changes in mass by altering their expenditure. Their flexibility to do this may be reduced as the absolute amount of food declines (because on average, expenditure must match intake when weight is stable), leading to a lower variance in day-to-day changes at higher levels of restriction. Whether this increased stability is an integral part of the life-extending impacts of CR remains unclear. The day-to-day variation in mass changes was not due to external factors like disturbance or room temperature changes because there was no indication of a positive correlation of changes among cages. Each mouse varied effectively at random through time relative to other mice in the same room.

Novel here is the demonstration that the regulatory ability (as reflected in the correlation with a lag of 1 day) declined with age in the mice fed AL and was significantly reduced in the last 50 days of life (in those individuals that died before the study ended) relative to their regulatory ability at the start of restriction. The reasons for this reduction in regulatory control with age in 12AL group remain unclear. Nevertheless, this is an interesting potential biomarker of regulatory decline as a reflection of aging. This is particularly so because, across all the mice that died prematurely, there was a significant reduction in regulatory control in the 50 days prior to their deaths. Interestingly, the decline with age was not observed in any of the groups of mice under CR up to the end of the study at age 24 months. This supports the general idea that CR retards the aging process and that this day-to-day regulatory feedback may be a useful biomarker of aging.

One question raised from our STCR studies was the extent to which a 3-month window can be used to characterize the responses of animals when exposed to CR for their entire lives. Here we reveal that the key changes in body composition occur in the first 30 days and are maintained thereafter with tight daily regulatory control. The unique data set allowed for in-depth analysis of the day-to-day body mass variation and supports the use of shorter-term CR studies.

## Supplementary Material

glad152_suppl_Supplementary_Materials

glad152_suppl_Supplementary_Tables
