# Supplementary material for: The Effects of Graded Levels of Calorie Restriction: XX. Impact of Long-Term Graded Calorie Restriction on Survival and Body Mass Dynamics in Male C57BL/6J Mice
Source: J Gerontol A Biol Sci Med Sci. 2023 Jun 24;78(11):1953–63. doi: 10.1093/gerona/glad152 (PMC10613020; doi:10.1093/gerona/glad152)
Supplement: glad152_suppl_Supplementary_Materials [file glad152_suppl_supplementary_materials.pdf]

Supplemental Materials

eFigure 1. Body composition changes in male C57BL/6J mice which underwent 580 days (19 months) of calorie restriction (CR).

eFigure 2. The relationship between body composition changes in male C57BL/6J mice under long term 588 days (19 months) graded calorie restriction (CR).

eFigure 3. Partial autocorrelation analysis of day to day of variation body mass.

eFigure 4. Relationship between daily body mass changes (Diff n) and changes with a lag of 1, 5 or 10 days.

eTable 1. Necroscopy findings determined from male C57BL/6J male mice over 585 days (19 month) graded calorie restriction (CR).

eTable 2. Details of necroscopy findings and lifespan of mice euthanised prior to the 24 month schedule timepoint.

eTable 3. Body composition of male C57BL/6J mice fed 12 hours ad libitum (12AL) or calorie restricted (CR) for 588 days (19 months).

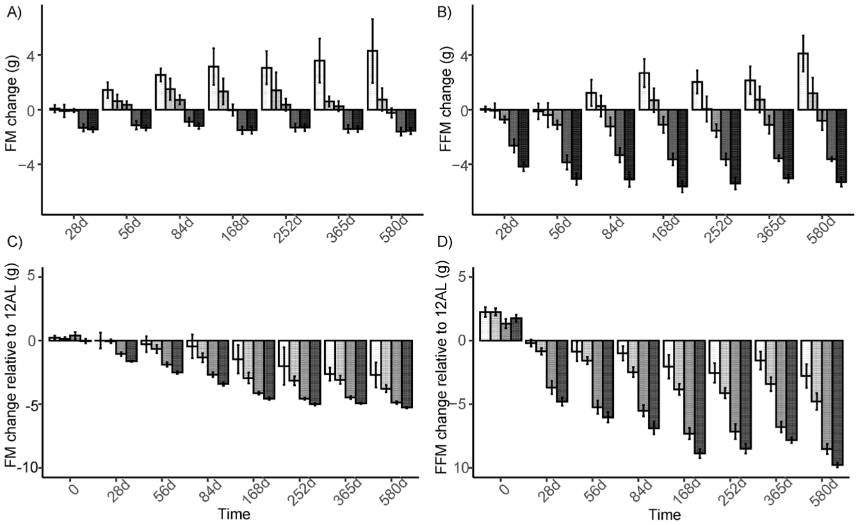

eFigure 1. Body composition changes in male C57BL/6J mice which underwent 580 days (19 months) of calorie restriction (CR). Mice were 728 days (24 months old at the end of study n=33. CR was graded from 10 to 40% (10CR, 20CR, 30CR, 40CR) based on baseline daily food intake. A control of 12AL was used where food was available *ad libitum* only over 12 hours of darkness. Body composition was measured by DXA and taken at 8 timepoints 0, 28, 56, 84, 168, 252, 365 and 580 days from initiation of CR. A) change in fat mass (FM) and B) fat free mass (FFM) compared to own baseline; C) and D) FM and FFM changes relative to 12AL control at each timepoint. Data shown as mean ± sd.

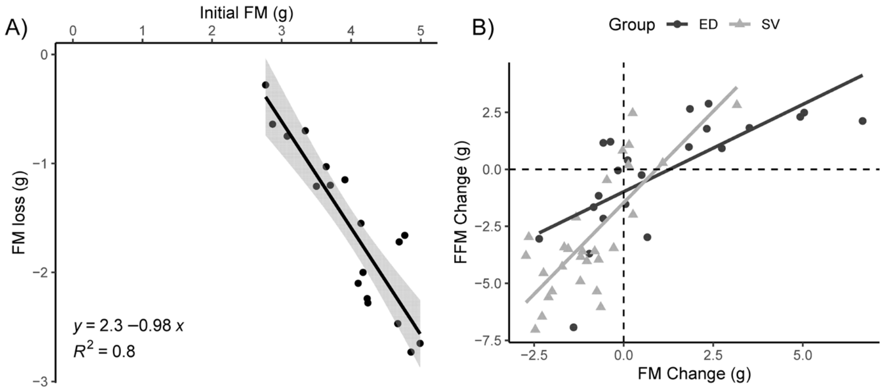

eFigure 2. The relationship between body composition changes in male C57BL/6J mice under long term 588 days (19 months) graded calorie restriction (CR). A) Loss of fat mass (FM) in relation to initial fat mass in male C57BL/6J mice restricted by 30% and 40% calorie restriction. B) FM and fat free mass (FFM) changes from baseline to time of death in 22 mice euthanased prior to the end of study (ED = early deaths) and SV = survivors, 28 mice aged 728 days (24 months at the end). Controls were not included.

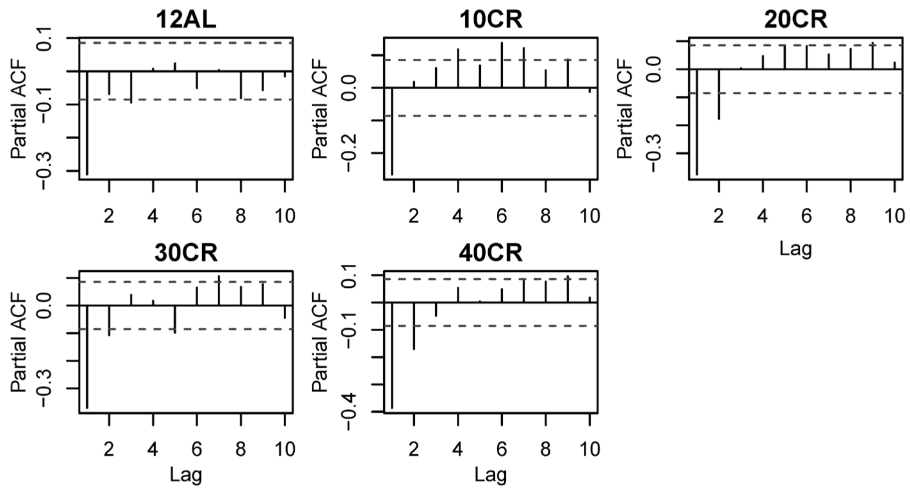

eFigure 3. Partial autocorrelation analysis of day to day of variation body mass.
Body mass changes were compared on day(n) to that on day(n+1), day(n+2), up to day(n+10).

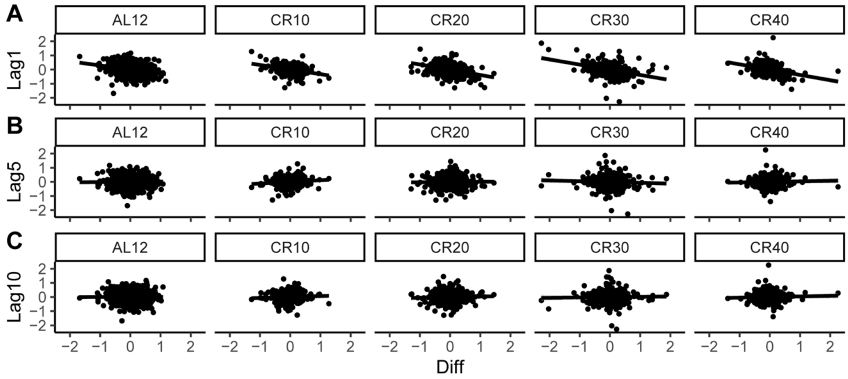

eFigure 4. Relationship between daily body mass changes (Diff n) and changes with a lag of 1, 5 or 10 days. Body mass difference separated by 1 day (Lag1 : A), 5 days (Lag5 : B) and 10 days (Lag10 : C). Examples are given of 1 mouse per group. Mice were fed 12 hrs *ad libitum* (12AL) or restricted by 10 20, 30 and 40 % of their individual baseline food intake (10CR, 20CR, 30CR and 40CR). Correlations are apparent only with a lag of 1.

eTable 1. Necroscopy findings determined from male C57BL/6J male mice over 585 days (19 month) graded calorie restriction (CR). Mice were restricted by 10, 20, 30 and 40% of baseline intakes (10CR, 20CR, 30CR and 40CR) or 12hr *ad libitum* feeding (12AL). Counts are provided for mice which were euthanised (Pre) and those that reached the 24 month end point (24m). Mice classed as 'Healthy' displayed no obvious disease states.

	12AL		10CR		20CR		30CR		40CR	
	**Pre**	**24m**	**Pre**	**24m**	**Pre**	**24m**	**Pre**	**24m**	**Pre**	**24m**
**Cancer / Neoplasia**	4	0	4	1	6	1	4*	3	1	4
**Age-related**	4	1	2*	1	0	0	0	0	1	2
**Non-Age-related**	1	0	4	0	0	0	0	0	0	1
**Healthy**	0	4	0	2	0	5	0	5	0	3
**Euthanised**	9	N/A	10	N/A	6	N/A	4	N/A	2	N/A
**Survivors**	N/A	5	N/A	4	N/A	6	N/A	8	N/A	10
**Total**	14		14		12		12		12	

eTable 2. Details of necroscopy findings and lifespan of mice euthanised prior to the 24 month schedule timepoint. HG: Hyperglycaemia, GTT: Glucose Tolerance Test, SV: Seminal Vesicle, SC: Subcutaneous

ID	Group	Vets advice for euthanasia	Necroscopy findings	Lifespan (days)
86	12AL	Swollen abdomen, suspected tumour	Liver tumour	472
51	12AL	HG post GTT / not eating		508
37	12AL	HG / not eating	Enlarged SV	511
45	12AL	HG post GTT / not eating. Lost >4g over 4 days	Enlarged SV	511
67	12AL	Erratic eating, swollen abdomen	Prostate tumour, enlarged SV & caecum	550
79	12AL	Erratic eating, starry	Liver tumour	556
94	12AL	Swollen abdomen	Necrotic SV	588
53	12AL	Lumps felt	SC fat tumour	704
59	12AL	Scruffy/ not eating	Discoloured SV / Pale liver	724
92	10CR	Suspected hernia	Hernia	255
48	10CR	Swollen abdomen	Liver tumour	561
84	10CR	Leaving food. Lump felt	SC fat tumour	588
36	10CR	Swollen abdomen	Distended intestines + Enlarged SV	645
85	10CR	Swollen abdomen	Distended, fused intestine + hardened epididymal fat	646
47	10CR	Not eating, scruffy, sore eye	Large, discoloured lungs	708
64	10CR	Not Eating. Abdominal lump felt	Intestinal Tumour	715
56	10CR	Abdominal lump felt	Necrotic SV	717
89	10CR	Not eating, abdominal lump	Liver & Intestinal Tumour	724
83	10CR	Found dead	Necrotic SV	725
42	20CR	Panting	Diaphragm tumour	459
57	20CR	Growth on mouth affecting eating/ losing weight	Mouth tumour	472
49	20CR	Leaving food. Abdominal lump	Prostate Tumour	560
93	20CR	Swollen abdomen/ scruffy	Liver tumour	583
95	20CR	Blood found in cage	SC fat tumour	623
61	20CR	Swollen abdomen	Liver tumour + intestinal problems	667
38	30CR	Found dead	Liver tumour	404
35	30CR	Swollen abdomen/ cold /stary	Liver tumour	456
87	30CR	Hard abdominal lump felt	Liver & SC fat tumour	634
88	30CR	Kyphosis	Liver tumour	684
66	40CR	Swollen abdomen	Intestinal tumour	679
55	40CR	Not Eating >30% weight loss		704

eTable 3. Body composition of male C57BL/6J mice fed 12 hours *ad libitum* (12AL) or calorie restricted (CR) for 588 days (19 months). Mice were restricted by 10%, 20%, 30% or 40% of their individual baseline food intakes. Body mass (BM), fat mass (FM), fat free mass (FFM) as measured by dual x-ray absorptiometry are shown as absolute weight at the end of study (24 months of age). The weight changes from baseline (BL) and the range in weight change, from lowest to highest, for each category are shown. Percentage changes relative to BL or the 12AL controls at end of study are shown. All data presented as average ± sd.

Group	Weight at end of study (g)	Weight change from BL (g)	Range (g)	Change relative to BL (%)	Realised change to 12AL (%)
**12AL**	BM: 39.22±8.2<br>FM: 8.09±4.9<br>FFM: 31.13±3.3	8.41 ± 8.1<br>4.30 ± 5.2<br>4.11 ± 2.9	2.36 to 21.46<br>0.18 to 12.53<br>1.65 to 8.93	27.39 ± 26.0<br>122.49 ± 145.1<br>15.20 ± 10.7	-<br>-<br>-
**10CR**	BM: 32.38±3.1<br>FM: 4.80±1.9<br>FFM: 27.58±1.9	1.94 ± 3.2<br>0.75 ± 1.6<br>1.19 ± 2.3	-1.73 to 5.98<br>-0.58 to -3.16<br>-1.99 to 2.82	6.41 ± 10.3<br>16.61 ± 36.8<br>4.64 ± 8.5	-17.45 ± 8.0<br>-40.74 ± 23.9<br>-11.39 ± 5.9
**20CR**	BM: 29.30±2.2<br>FM: 3.72±0.7<br>FFM: 25.58±1.6	-1.05 ± 2.4<br>-0.24 ± 0.8<br>-0.81 ± 1.7	-4.4 to 1.37<br>-1.33 to 1.09<br>-3.59 to 0.82	-3.39 ± 7.8<br>-5.13 ± 21.4<br>-3.03 ± 6.4	-25.29 ± 5.5<br>-54.05 ± 8.6<br>-17.81 ± 5.1
**30CR**	BM:24.48±1.3<br>FM: 2.63±0.3<br>FFM: 21.85±1.1	-5.22 ± 0.8<br>-1.6 ± 0.8<br>-3.63 ± 0.4	-6.52 to -3.73<br>-2.73 to -0.28<br>-4.27 to -2.97	-17.58 ± 2.7<br>-35.75 ± 14.5<br>-14.26 ± 1.7	-37.59 ± 3.4<br>-67.56 ± 3.9<br>-29.8 ± 3.6
**40CR**	BM: 22.83±0.6<br>FM: 2.23±0.2<br>FFM: 20.60±0.6	-6.87 ± 1.5<br>-1.56 ± 0.7<br>-5.31 ± 1.0	-9.5 to -4.66<br>-2.47 to -0.64<br>-7.03 to -3.84	-23.0 ± 4.2<br>-39.34 ± 13.8<br>-20.42 ± 3.4	-41.80 ± 1.7<br>-72.43 ± 2.7<br>-33.84 ± 1.8
