# Supplementary material for: The Effects of Graded Levels of Calorie Restriction: XX. Impact of Long-Term Graded Calorie Restriction on Survival and Body Mass Dynamics in Male C57BL/6J Mice
Source: J Gerontol A Biol Sci Med Sci. 2023 Jun 24;78(11):1953–63. doi: 10.1093/gerona/glad152 (PMC10613020; doi:10.1093/gerona/glad152)
Supplement: glad152_suppl_Supplementary_Tables [file glad152_suppl_supplementary_tables.docx]

**Supplementary Table 1**. Necroscopy findings determined from male C57BL/6J male mice over 585 days (19 month) graded calorie restriction (CR) at 10, 20, 30 and 40% of baseline intakes (10CR, 20CR, 30CR and 40CR) or 12hr *ad libitum* feeding (12AL). Counts are provided for mice which were euthanased (Pre) and those that reached the 24 month end point (24m). Mice classed as ‘Healthy’ displayed no obvious disease states.

|  | **12AL** | | **10CR** | | **20CR** | | **30CR** | | **40CR** | |
| --- | --- | --- | --- | --- | --- | --- | --- | --- | --- | --- |
|  | **Pre** | **24m** | **Pre** | **24m** | **Pre** | **24m** | **Pre** | **24m** | **Pre** | **24m** |
| **Cancer / Neoplasia** | 4 | 0 | 4 | 1 | 6 | 1 | 4* | 3 | 1 | 4 |
| **Age-related** | 4 | 1 | 2* | 1 | 0 | 0 | 0 | 0 | 1 | 2 |
| **Non-Age-related** | 1 | 0 | 4 | 0 | 0 | 0 | 0 | 0 | 0 | 1 |
| **Healthy** | 0 | 4 | 0 | 2 | 0 | 5 | 0 | 5 | 0 | 3 |
| **Euthanased** | 9 | N/A | 10 | N/A | 6 | N/A | 4 | N/A | 2 | N/A |
| **Survivors** | N/A | 5 | N/A | 4 | N/A | 6 | N/A | 8 | N/A | 10 |
| **Total** | 14 | | 14 | | 12 | | 12 | | 12 | |

*Indicates the two mice which were found dead naturally over the course of the study.

**Supplementary Table 2 Details of necroscopy findings and lifespan of mice euthanased prior to the 24 month schedule timepoint.**

| **ID** | **Group** | **Vets advice for euthanasia** | **Post mortem findings** | **Lifespan (days)** |
| --- | --- | --- | --- | --- |
| 86 | 12AL | Swollen abdomen, suspected tumour | Liver tumour | 472 |
| 51 | 12AL | HG post GTT / not eating |  | 508 |
| 37 | 12AL | HG / not eating | Enlarged SV | 511 |
| 45 | 12AL | HG post GTT / not eating. Lost >4g over 4 days | Enlarged SV | 511 |
| 67 | 12AL | Erratic eating, swollen abdomen | Prostate tumour, enlarged SV & caecum | 550 |
| 79 | 12AL | Erratic eating, starry | Liver tumour | 556 |
| 94 | 12AL | Swollen abdomen | Necrotic SV | 588 |
| 53 | 12AL | Lumps felt | SC fat tumour | 704 |
| 59 | 12AL | Scruffy/ not eating | Discoloured SV / Pale liver | 724 |
| 92 | 10CR | Suspected hernia | Hernia | 255 |
| 48 | 10CR | Swollen abdomen | Liver tumour | 561 |
| 84 | 10CR | Leaving food. Lump felt | SC fat tumour | 588 |
| 36 | 10CR | Swollen abdomen | Distended intestines + Enlarged SV | 645 |
| 85 | 10CR | Swollen abdomen | Distended, fused intestine + hardened epididymal fat | 646 |
| 47 | 10CR | Not eating, scruffy, sore eye | Large, discoloured lungs | 708 |
| 64 | 10CR | Not Eating. Abdominal lump felt | Intestinal Tumour | 715 |
| 56 | 10CR | Abdominal lump felt | Necrotic SV | 717 |
| 89 | 10CR | Not eating, abdominal lump | Liver & Intestinal Tumour | 724 |
| 83 | 10CR | Found dead | Necrotic SV | 725 |
| 42 | 20CR | Panting | Diaphragm tumour | 459 |
| 57 | 20CR | Growth on mouth affecting eating/ losing weight | Mouth tumour | 472 |
| 49 | 20CR | Leaving food. Abdominal lump felt. | Prostate Tumour | 560 |
| 93 | 20CR | Swollen abdomen/ scruffy | Liver tumour | 583 |
| 95 | 20CR | Blood found in cage | SC fat tumour | 623 |
| 61 | 20CR | Swollen abdomen | Liver tumour + intestinal problems | 667 |
| 38 | 30CR | Found dead | Liver tumour | 404 |
| 35 | 30CR | Swollen abdomen/ cold /stary | Liver tumour | 456 |
| 87 | 30CR | Hard abdominal lump felt | Liver & SC fat tumour | 634 |
| 88 | 30CR | Kyphosis | Liver tumour | 684 |
| 66 | 40CR | Swollen abdomen | Intestinal tumour | 679 |
| 55 | 40CR | Not Eating >30% weight loss |  | 704 |

**Supplementary Table 3** Body composition of male C57BL/6J mice fed 12 hours *ad libitum* (12AL) or calorie restricted (CR) for 588 days (19 months) by 10%, 20%, 30% or 40% of their individual baseline food intakes. Body mass (BM), fat mass (FM), fat free mass (FFM) as measured by dual x-ray absorptiometry are shown as absolute weight at the end of study (24 months of age). The weight changes from baseline (BL) and the range in weight change, from lowest to highest, for each category are shown. Percentage changes relative to BL or the 12AL controls at end of study are shown. All data presented as average ± sd.

| **Group** | **Weight at end of study (g)** | **Weight change from BL (g)** | **Range (g)** | **Change relative to BL (%)** | **Realised change to 12AL (%)** |
| --- | --- | --- | --- | --- | --- |
| **12AL** | BM: 39.22±8.2  FM: 8.09±4.9  FFM: 31.13±3.3 | 8.41 ± 8.1  4.30 ± 5.2  4.11 ± 2.9 | 2.36 to 21.46  0.18 to 12.53  1.65 to 8.93 | 27.39 ± 26.0  122.49 ± 145.1  15.20 ± 10.7 | -  -  - |
| **10CR** | BM: 32.38±3.1  FM: 4.80±1.9  FFM: 27.58±1.9 | 1.94 ± 3.2  0.75 ± 1.6  1.19 ± 2.3 | -1.73 to 5.98  -0.58 to -3.16  -1.99 to 2.82 | 6.41 ± 10.3  16.61 ± 36.8  4.64 ± 8.5 | -17.45 ± 8.0  -40.74 ± 23.9  -11.39 ± 5.9 |
| **20CR** | BM: 29.30±2.2  FM: 3.72±0.7  FFM: 25.58±1.6 | -1.05 ± 2.4  -0.24 ± 0.8  -0.81 ± 1.7 | -4.4 to 1.37  -1.33 to 1.09  -3.59 to 0.82 | -3.39 ± 7.8  -5.13 ± 21.4  -3.03 ± 6.4 | -25.29 ± 5.5  -54.05 ± 8.6  -17.81 ± 5.1 |
| **30CR** | BM:24.48±1.3  FM: 2.63±0.3  FFM: 21.85±1.1 | -5.22 ± 0.8  -1.6 ± 0.8  -3.63 ± 0.4 | -6.52 to -3.73  -2.73 to -0.28  -4.27 to -2.97 | -17.58 ± 2.7  -35.75 ± 14.5  -14.26 ± 1.7 | -37.59 ± 3.4  -67.56 ± 3.9  -29.8 ± 3.6 |
| **40CR** | BM: 22.83±0.6  FM: 2.23±0.2  FFM: 20.60±0.6 | -6.87 ± 1.5  -1.56 ± 0.7  -5.31 ± 1.0 | -9.5 to -4.66  -2.47 to -0.64  -7.03 to -3.84 | -23.0 ± 4.2  -39.34 ± 13.8  -20.42 ± 3.4 | -41.80 ± 1.7  -72.43 ± 2.7  -33.84 ± 1.8 |
